# Performance Analysis of Deep-Neural-Network-Based Automatic Diagnosis of Diabetic Retinopathy

**DOI:** 10.3390/s22010205

**Published:** 2021-12-29

**Authors:** Hassan Tariq, Muhammad Rashid, Asfa Javed, Eeman Zafar, Saud S. Alotaibi, Muhammad Yousuf Irfan Zia

**Affiliations:** 1 Department of Electrical Engineering, School of Engineering, University of Management and Technology (UMT), Lahore 54770, Pakistan; hassantariq@umt.edu.pk (H.T.); asfa.javed@umt.edu.pk (A.J.); f2018019042@umt.edu.pk (E.Z.); 2 Department of Computer Engineering, Umm Al-Qura University, Makkah 21955, Saudi Arabia; mfelahi@uqu.edu.sa; 3 Department of Information Systems, Umm Al-Qura University, Makkah 21955, Saudi Arabia; ssotaibi@uqu.edu.sa; 4 Telecommunications Engineering School, University of Malaga, 29010 Malaga, Spain

**Keywords:** deep learning, diabetic retinopathy, deep transfer learning, convolutional neural network, automatic detection

## Abstract

Diabetic retinopathy (DR) is a human eye disease that affects people who are suffering from diabetes. It causes damage to their eyes, including vision loss. It is treatable; however, it takes a long time to diagnose and may require many eye exams. Early detection of DR may prevent or delay the vision loss. Therefore, a robust, automatic and computer-based diagnosis of DR is essential. Currently, deep neural networks are being utilized in numerous medical areas to diagnose various diseases. Consequently, deep transfer learning is utilized in this article. We employ five convolutional-neural-network-based designs (AlexNet, GoogleNet, Inception V4, Inception ResNet V2 and ResNeXt-50). A collection of DR pictures is created. Subsequently, the created collections are labeled with an appropriate treatment approach. This automates the diagnosis and assists patients through subsequent therapies. Furthermore, in order to identify the severity of DR retina pictures, we use our own dataset to train deep convolutional neural networks (CNNs). Experimental results reveal that the pre-trained model Se-ResNeXt-50 obtains the best classification accuracy of 97.53% for our dataset out of all pre-trained models. Moreover, we perform five different experiments on each CNN architecture. As a result, a minimum accuracy of 84.01% is achieved for a five-degree classification.

## 1. Introduction

Diabetic retinopathy (DR) is a human eye infection in people with diabetes. It is initiated due to retinal vascular damage, which is caused by diabetes mellitus for a long-duration [1]. This disease is one of the most common reasons behind blindness [2]. Therefore, its detection in the early stages is critical [3]. There are many treatments for this disease; however, they take plenty of time and may even include many eye tests such as photo-coagulation and vitrectomy [4].

According to a survey in Europe, almost 60 million people are diabetes patients and they are most prone to DR. In the United States, 10.2 million people with an age of 40 or above have diabetes. Furthermore, 40% of these people are at risk of some vision-threatening disease [5]. Moreover, the survey of the Center for Disease Control in 2020 revealed that 3.3 million people are suffering from DR [6]. According to the World Health Organization, diabetes has affected 422 million people to date and this number will become 629 million by 2045 [7,8].

DR is normally categorized into five different groups: Normal-0, Mild-1, Moderate-2, Severe-3 and Proliferative-4 as listed in Table 1. The disease starts with small changes in the blood vessels of the eyes, which could be labeled as Mild DR. Concerning the case of Mild DR, the patient could defeat this disease and complete recovery is possible. If this condition of the disease is left untreated, then it will convert into Moderate DR. The leakage in the blood vessels may start in the case of Moderate DR. In the next stage, if the disease increases further then it changes to Severe and Proliferative DR and it could cause complete vision loss.

The current detection of DR is made through a dilated eye exam in which the doctors put some eye drops into the patient’s eyes. Subsequently, an image of the eye is taken with the help of various medical instruments. This technique is manual and therefore there are always some errors in diagnosis. Another way of detecting DR is examining through ophthalmoscopy. In one study, approximately 16% of patients were diagnosed as DR patients using ophthalmoscopy in respect of 442 right eyes [10].

Image processing is also used to identify DR based on highlights; for example, veins, radiates, hemorrhages and small-scale aneurysms. During this process, digital fundus cameras are used to obtain accurate eye images. Techniques like image enhancement, fusion, morphology detection and image segmentation help medical doctors to obtain more information from the data of medical images [10]. In the case of DR, people are not aware of the disease unless a manual detection is made. Due to the lack of related treatment, according to the specific level of the disease, chances of losing eyesight may increase [11].

### 1.1. State-of-the-Art on DR Detection Dsing Deep Learning Techniques

Numerous techniques have been proposed to detect DR. This section focuses on multi-class classification using deep learning and neural network techniques. Some studies have classified the fundus images into two categories: diabetic, which includes average to extreme conditions of non-proliferative DR; and non-diabetic, where the person is not affected with DR) [12]. Based on this, they proposed a technique to accurately appoint the class where a fundus image could be labeled, utilizing one principal classifier and back propagation neural organization (BPNN) procedures.

Similarly, a deep-learning-based method has been proposed to classify the fundus photographs for human ophthalmologist diagnostics. Authors have built a novel Siamese-like CNN (convolutional neural network) binocular model based on Inception V3 that can acknowledge fundus pictures of both eyes and yield the output of each eye at the same time [13]. A hybrid approach for diagnosing DR has been proposed that uses histogram equalization (HE) and contrast limited adaptive histogram equalization (CLAHE) to assist the deep learning model [14]. It provides more accentuation and effectiveness by way of the intelligent enhancement of the image during the diagnosis process. The authors exploited five CNN architectures to evaluate the performance parameters for the dataset of DR patients. Their classification methodology classifies images into three different groups based on the condition of the disease [15].

The authors developed a novel ResNet18-based CNN architecture to diagnose DR patients. This approach helps in solving a strong class imbalance problem and generates region scoring maps (RSMs) [16]. Furthermore, it indicates the severity level by highlighting the semantic regions of the fundus image. The authors proposed a technique only for the detection of DR regardless of the severity of DR. They classified images as normal and abnormal for the targeted dataset [17]. Similarly, the authors proposed a deep-learning-based CNN to classify a small dataset of DR images, using Cohen’s kappa as an accuracy metric [18].

In addition to the aforementioned research works, many datasets of fundus images have been developed for DR-related diagnoses. For example, TeleOphta uses a tele-ophthalmology network for diabetic retinopathy screening [19]. Other examples are Digital Retinal Images for Vessel Extraction (DRIVE) and Structured Analysis of the Retina (STARE), which are used to segment the vessel network using local and global vessel features [20,21]. Similarly, the SVM (support vector machine) provides 95% and Bayesian provides 90% accuracy [11]. In this technique, images are segmented, outliers are detected, image analysis is performed and the brightness is controlled. In an another technique, SVM provides 86% accuracy and KNN (K-nearest neighbor) provides 55% accuracy [22]. In KNN, images are clustered with the help of pixel clusters. The fundus image mask is removed with the help of pixel clustering [22].

There is another technique known as the extreme learning machine (ELM) design for detecting a disease in eye blood vessels. This technique is mainly used for the detection of diseased blood vessels. Some of the blood vessels are injured in diabetic retinopathy. In this technique, an image is provided to the ELM. The provided algorithm calculates the grayscale value and chooses some features that provide more information than other pixels. Consequently, researchers can achieve 90% accuracy [10]. Similarly, the authors analyzed various blood vessel segmentation techniques in [23,24]. They further identified the lesions for the detection of diabetic retinopathy. The results were compared with the neural network technique.

Finally, by integrating microaneurysms, haemorrhages and exudates, the authors described a method for detecting non-proliferative diabetic retinopathy [25]. They developed a novel convolutional layer that automatically determines the number of extracted features. Each category is then placed into different folders so that there exist a small number of patches for the model to process at runtime. Subsequently, six convolutional layers are added to the model to obtain a validation accuracy of 72% and a training accuracy of 75% .

### 1.2. Research Gap

Although pre-trained CNNs have been used previously for different diseases, there is a need to enhance the accuracy of classification using a custom dataset and deep transfer learning. A dataset composed of low-resolution DR images, as employed in the conventional methods of Section 1.1, may cause low accuracy or incorrect classification. At the same time, a high-risk patient in the proliferate category requires immediate cure and diagnosis. Keeping this view, the diagnosis procedure requires high accuracy with adequate images of the posterior pole. In a nutshell, there should be an efficient, immediate and autonomous method that can recognize retinopathy with accurate outcomes. This implies that there should be a methodology to evaluate the classification performance parameters on recent CNN architectures.

### 1.3. Contributions

In this article we propose a methodology to classify the DR images using five different pre-trained CNNs. The contributions are summarized in the following points:

- Our proposed methodology is flexible and automatically detects the classified pictures of patients with a higher accuracy. It classifies the dataset based on the severity of the disease in different stages/categories. Moreover, it helps doctors to select one or more CNN architectures for the diagnosis.
- We have analyzed the robustness of CNN architectures on our constructed (customized) dataset for the diagnosis of DR patients. A brief description of the customized dataset is provided in Section 1.4. It highlights how both CNN and dataset directly or indirectly affect performance evaluation. It implies that deep transfer learning techniques have been used with some pre-trained models and customized datasets to obtain high-accuracy results.
- We have also analyzed how the previously made architectures will perform on our dataset and how these architectures can be fine-tuned to obtain the best results on our dataset.
- To the best of our knowledge, the proposed work in this article is the first effort to consider the evaluation of recent CNNs, using a customized dataset.

The objective is to provide accurate and less time-consuming results (as compared to the manual methods) by applying different deep neural network algorithms for the classification of different eyes infected by the illness. This helps to obtain more information from the classified images. Consequently, doctors will be able to detect diabetic retinopathy levels more accurately.

### 1.4. Customized Dataset for Performance Evaluation

The classification accuracy of the DR mainly depends upon the size of the dataset. This implies that a higher accuracy requires a huge amount of training data using a machine learning algorithm. Moreover, the data should be collected from reliable sources with accurate tags. The following datasets are most widely used for DR detection: Digital Retinal Images for Vessel Extraction (DRIVE) dataset [20], Structured Analysis of the Retina (STARE) dataset [21], E-ophtha dataset [19] and Kaggle Diabetic Retinopathy dataset [26,27].

In this study, we created our custom dataset as explained in Section 4.1. The created dataset was built from different resources which are based on different severity levels. It also includes EyePacs [26], which has collected approximately 5 million images from 75,000 patients. Another dataset from Kaggle, which consists of 53,594 images for testing and 35,126 images for training, is also available for analysis. The Kaggle dataset includes a significant number of pictures (72,743) from DR patients. Furthermore, it has pictures for all DR categories in a single folder. Moreover, it also contains categories of various images and their descriptions in the form of comma separated value (CSV) files.

The corresponding enhancements and preprocessing of data are explained in Section 2.1, where all the images are oriented, resized and horizontally flipped. Moreover, the intensity of images is also enhanced.Furthermore, an augmentation is performed where all the images are made consistent in terms of size and intensity. The aforementioned enhancements and preprocessing techniques help CNN for the robust classification. Based on the aforementioned databases, we constructed a dataset of 5333 images, where 1421 are normal, 954 are mild, 1210 are moderate, 308 are severe and 1440 are high-risk patients (see Section 4.1).

### 1.5. Organization

The organization of this paper is as follows. The proposed methodology is described in Section 2. Section 3 explains the pre-trained CNN architectures and different performance matrices used in the results. Section 4 reports the results and implementation in light of the proposed methodology. The article is concluded in Section 5.

## 2. Proposed Approach

**Figure 1:**
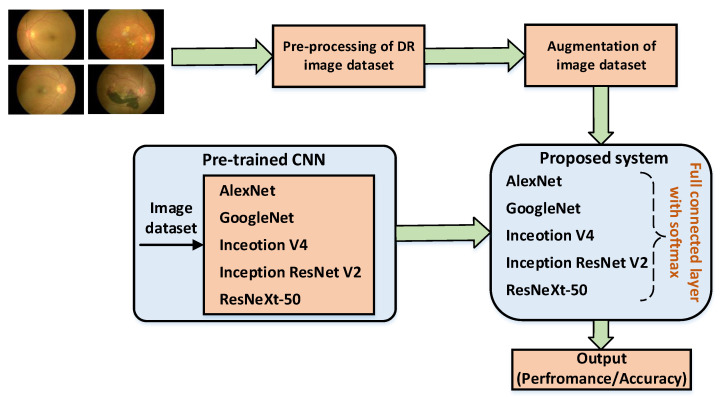
Proposed framework for the detection of DR.

The proposed methodology is illustrated in Figure 1. The entire process comprises five steps. First, the retina pictures are pre-processed and supplemented using pre-trained models. Deep transfer learning (DTL) is then used during the training phase. During classification, feature extraction and precise prediction of models are employed. The retina prediction is made using a machine learning algorithm. Subsequently, it is classified into five different groups based on the severity of DR as described in Table 1.

The following steps are involved during the prediction process: dataset, data pre-processing, model setup and evaluation. In the dataset, the method of data generation for training and testing purposes is described. In data pre-processing, the pipeline for bringing the pictures from various sources is portrayed. Similarly, the model setup describes multiple convolution layers for the classification of images. Finally, the results are evaluated and analyzed.

The data were collected from different resources to construct a new dataset. Furthermore, Python visualization libraries were used to visualize our data [27]. The proposed method in this article employs deep neural networks and a supervised learning architecture (CNN) for image detection. The supervised learning is used for model training. After model training, the sample data are tested and verified with the given training data. Moreover, some evaluation techniques are applied for the classification of results. After executing classification techniques, results are classified on the basis of training data. Finally, the model accuracy is measured in comparison to training data.

### 2.1. Pre-Processing and Enhancement of DR Dataset

Actual pre-trained CNN models are too large to handle the retina images dataset, resulting in overfitting issues. To address this problem, a variation can be introduced to the dataset. Adding variation at the early point (input) of a neural network causes significant changes in the dataset generalization. A variation refers to the fact that the noise addition task augments the dataset in some way. The dataset constraint is one of the critical challenges faced by researchers in the healthcare field. As a result, we have employed some additional augmentation approaches. The retina image dataset was created as follows. After resizing the photos to 224 × 224 × 3, we used the following augmentation methods: random horizontal flip (aids in the detection of DR based on severity level), random resized crop (the last stage of DR, i.e., proliferate) and, last, picture enhancement by altering picture intensities.

### 2.2. CNN Architecture

**Figure 2:**
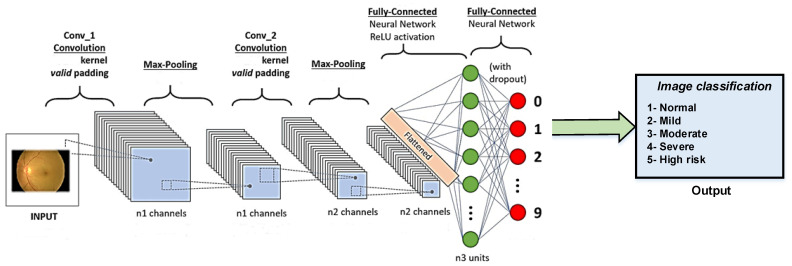
Schematics of CNN model for the detection of different DR stages.

Deep neural networks based on CNN models have recently been employed to handle computer vision challenges. To categorize the DR dataset among normal and various levels of DR patients, we employed deep-CNN-model-based AlexNet [28], GoogleNet [29], Inception V4 [30], Inception ResNet V2 [31] and ResNeXt-50 [32] models, as well as transfer learning approaches. Transfer learning may also aid with class imbalance and model execution time. The employed CNN models, as well as AlexNet, GoogleNet, Inception V4, Inception ResNet V2 and ResNeXt-50 models, are presented schematically in Figure 2. Pre-trained models work quite well on a new dataset before being used for classification.

**Figure 3:**
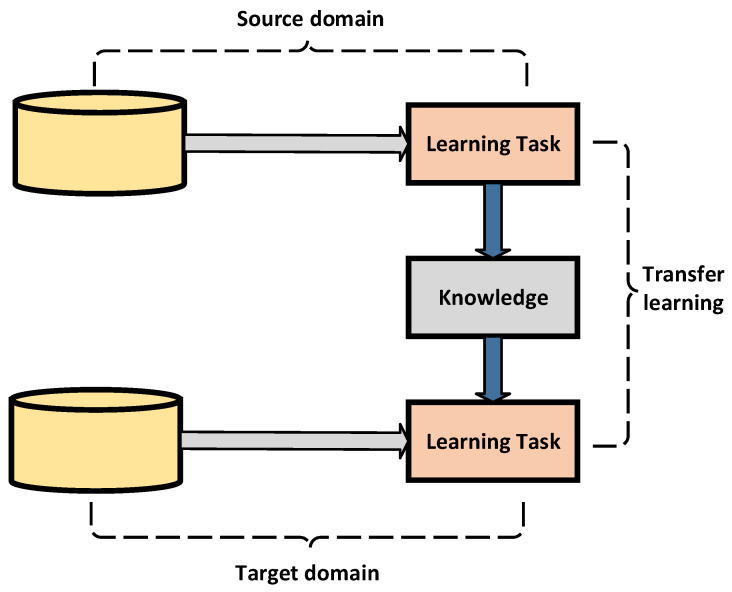
The adopted DTL process.

The DTL is a useful approach for solving the issue of unfit training data. The goal of this strategy is to extract the information from a process (issue). The extracted information is then utilized over comparable tasks by overcoming isolated learning issues. This understanding provides an incentive to tackle the problems in a variety of disciplines where the development is hard. It has resulted in insufficient or partial training data. Figure 3 depicts the DTL process.

**Figure 4:**
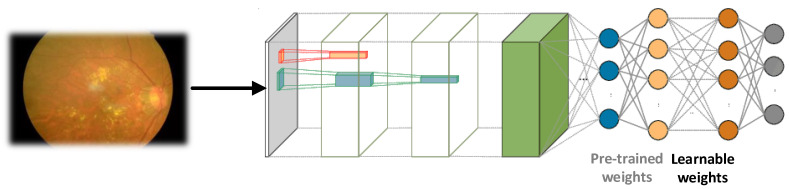
The DTL with pre-trained and learnable weights.

We utilize nine pre-trained architectures to deal with the retina image dataset, rather than using the long training process from scratch. The weights of existing pre-trained model layers are re-used for model training in a different domain, as illustrated in Figure 4. The DTL methodology has yielded beneficial and significant achievements in a variety of computer vision areas [33,34,35,36]. We used CNN architectural weights that had already been learned. Moreover, the entire model was fine-tuned with some appropriate learning rates.

## 3. Pre-Trained CNN Architectures and Performance Matrices

We selected five distinct pre-trained CNN architectures: AlexNet [28], GoogleNet [29], Inception V4 [30], Inception ResNet V2 [31] and ResNeXt-50 [32]. These models are used to classify the DR image dataset. In order to modify the classification layer, fine tuning is employed. The fine-tuning process extracts features for the targeted tasks. Since pre-trained models are utilized, only the previously diagnosed diabetic retinopathy images are used to make the models more accurate. The model training process is given as follows:

- Load the pictures from every type of folder.
- Use cv2 to resize images in (80, 80) and transmit images to array.
- Label every picture with type.
- Transform pictures and labels to numpy array.
- Split the images in half, and in an 80–20 split, the labels change into category labels.
- Set parameters of the trained model (e.g., epochs = 100, batch size = 32, etc.).
- Pickle may be used to save both the model and the label.
- In the end, we can visualize loss and accuracy.

### 3.1. AlexNet Architecture

**Figure 5:**
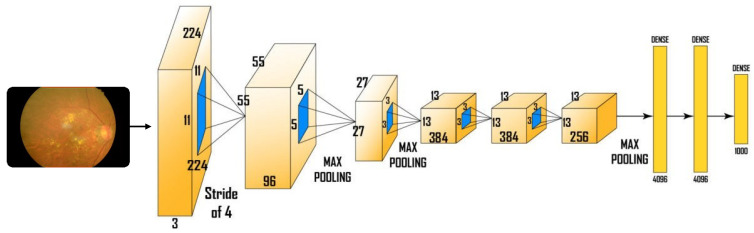
The pre-trained architecture of AlexNet.

AlexNet is the name of a convolutional neural network that has made a significant contribution to the field of machine learning. This is particularly true for in-depth learning in machine vision. The AlexNet architecture has 5 layers of flexibility, 3 layers of merging, 2 layers of standardization, 2 fully connected layers, and 1 softmax tile. The convolutional filters and the nonlinear activation function ReLU are included in each convolutional layer. Blending layers are used to create a variety of combinations. Due to the existence of completely linked layers, the input size is modified. Convolutional neural networks are a key component of neural networks. They are made up of neurons with a readable weight and bias. Each specific neuron receives a number of inputs. Subsequently, it takes a weight-bearing amount on top of it. Finally, it is transmitted by activating, turning and releasing. The complete architecture of AlexNet is illustrated in Figure 5.

### 3.2. GoogleNet Architecture

**Figure 6:**
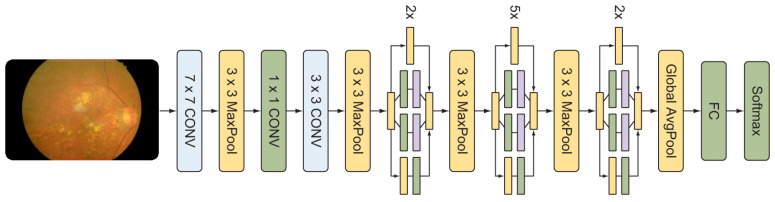
The pre-trained architecture of GoogleNet.

GoogleNet is a 22-level deep congenital neural network. Its salient feature is to work very fast. It has less memory usage and less power consumption. This neural network utilizes the averaged value of global pooling and maximum pooling. For our pre-trained model, it consists of four parallel paths. The inception blocks perform a convolution (1 × 1, 3 × 3, 5 × 5 window sizes) for spatial sizes and information extraction. The ReLU is also included in the convolution layer. The inception block is utilized three times. The first two inception blocks are used for 3 × 3 maximum pooling, while the third is used as a global average pool linked by a thick layer. The complete architecture of GoogleNet is illustrated in Figure 6.

### 3.3. Inception V4 Architecture

**Figure 7:**
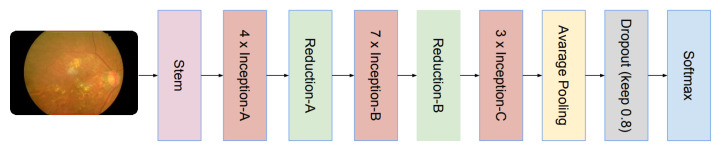
The pre-trained architecture of Inception V4.

Concerning the deep CNN architectures, Inception is considered for a good performance with a low execution cost. It was initially introduced in [31] as Inception v1. Then, this architecture was improved with the concept of batch normalization to a new variant named Inception v2. Next, factorization was introduced during iterations to form different variants, i.e., Inception v4, Inception ResNet V1 and Inception ResNet V2. Inception v4 is a slightly modified version of Inception v3. The Inception model and Inception ResNet model are residual and non-residual variants; this is the main difference. Moreover, batch normalization is only used on top of the traditional layer rather than residual summations. The architecture of Inception v4 consists of the initial set of layers that were modified to make it uniform. This is referred to as the “stem of the architecture” and is used in front of the Inception block in the architecture. This does not require the partition of the replicas, which enables a training feature. However, the previous versions of the Inception architecture require a replica to fit in the memory. This also reduces the memory requirement because it uses memory optimization during backpropagation. In our paper, we use Inception v4 and Inception ResNet V2. The explanation of Inception ResNet V2 is given in the next section. The complete architecture of Inception V4 is illustrated in Figure 7.

### 3.4. Inception ResNet V2 Architecture

**Figure 8:**
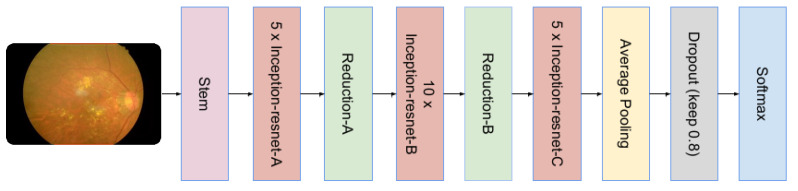
The pre-trained architecture of Inception ResNet V2.

Inception ResNet V2 is a decisive neural structure built into the Inception family of architectures. It incorporates residual connections (changes the filter concatenation stage of Inception construction). It has an ability to split images into 1000 objects, e.g., mouse, keyboard, pencil. The network has a read rich property to accept presentations of various images. The network has an input image size of 299 × 299. The output is a vector form of measurable probability. The complete build of the network is based on a combination of the original structure and the remaining connections. Moreover, multiple heavy filters are integrated with the remaining connections. The use of residual connections not only prevents degradation (caused by deep structures) but also reduces the training time. The complete architecture of Inception ResNet V2 is illustrated in Figure 8.

### 3.5. ResNeXt-50 Architecture

**Figure 9:**
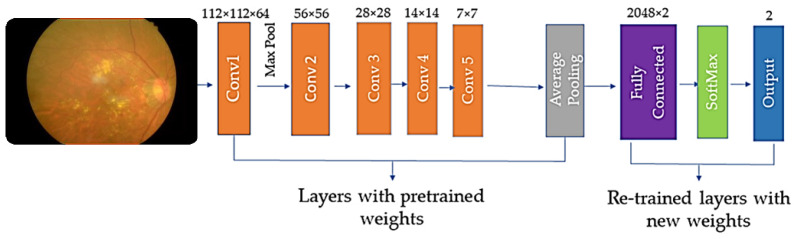
The pre-trained architecture of ResNeXt-50.

ResNeXt-50 uses a squeeze and excitation (SE) block for each non-identity branch of a residual block. It comprises 5 different sections, including convolution and identity blocks. A single convolution block has three layers of convolution and each ID has 3 stages of conversion. The SE block acts as a computational unit that performs transformations from inputs to feature maps. It can be attached with different CNN architectures and residual networks. The SE block is placed before summation, which increases the computational cost. However, it enables ResNext-50 to achieve a higher accuracy as compared to ResNet-50. The complete architecture of Inception ResNet V2 is illustrated in Figure 9.

### 3.6. Performance Matrices

All of the previously mentioned CCNs were utilized in our experiments. We considered five parameters to evaluate the aforementioned CNN architectures to classify the retina images. All these parameters were calculated using four important terms from the confusion matrix, which are True Positive (TP), True Negative (TN), False Positive (FP) and False Negative (FN). Therefore, the corresponding values for these parameters (accuracy, error rate, precision, recall and Fscore) are given in Equations (1) and (4)–(6), subsequently.

(1) $$Accuracy=\frac{TP+TN}{FN+TP+TN+FP}$$

(2) $$Recall/Sensitivity=\frac{TP}{TP+FN}$$

(3) $$Specificity=\frac{TN}{TN+FP}$$

(4) $$Precision=\frac{TP}{TP+FP}$$

(5) $$Error=\frac{1}{N}\sum_{n=1}^{j}\left|y_{j}-\hat{y_{j}}\right|$$

(6) $$Recall=2\times \left\{\frac{Precision\times Recall}{Precision+Recall}\right\}$$

The accuracy of the classifier depends on different parameters, as given in Equation (1). Moreover, the rate of sensitivity interprets the ability of a classifier to correctly form the target class, as given in Equation (2). Similarly, the rate of specificity illustrates the capability of a classifier for separation, as shown in Equation (3). The precision rate evaluates the determination of a certain class. Finally, FScore is the harmonic mean sensitivity (recall) and accuracy value as set forth in Equation (6). The analytical average error value may be determined using Equation (5). In our research, all associated evaluation parameters for CNNs were computed. Consequently, the findings are presented in the next section based on the above parameters.

## 4. Results and Implementation

This section provides the description of the proposed custom dataset, experimental setup and obtained results.

### 4.1. Creation of Custom Dataset

We created our custom dataset of fundus images to grade the severity level of DR. The proposed approach contrasts with the existing grading (as mentioned in Section 1.1), which grades fundus images based on the pathological changes in the retina. In addition to this, we consider the clinical practice; that is, we categorize a fundus picture of the foundation of abnormalities and the treatment technique. For training and testing, the pictures are divided and placed in different files. A custom script is created to determine the kind of picture based on its tags. The pictures are then cropped and the essential characteristics are separated. Furthermore, a filtering technique is employed to equalize and contrast the picture modification. To increase the variety of data, data augmentation is used. Finally, flipping, cropping and padding are performed. To summarize, the created dataset comprises 1440 images for positive DR patients (high-risk).

### 4.2. Experimental Setup

We developed some fine-tuned CNN architectures to classify DR pictures. These architectures are AlexNet, GoogleNet, Inception V4, Inception ResNet V2 and ResNeXt-50. Each CNN architecture uses fully connected (FC) layers with a classification criticality of the final FC layer. The number of neurons in the final FC layer is calculated using the target dataset. It is necessary to set and optimize these parameters as the CNN architecture itself is not able to define parameters for the fine-tuning method. Therefore, the parameters are defined using the results of training for the improvement of performance. We utilized the Adam optimizer for the training of every network architecture with 30 epochs (maximum). The batch size and initial learning rate for training and testing are 32, 8 and 1e-5, respectively. We used Python (a programming language) to train the CNN models. All the experiments were executed on an NVIDIA GPU (NVIDIA CUDA Version: 10.1 with Tesla P100) using a Google Colaboratory. PyTorch version 1.5 was utilized to execute experimentation on pretrained CNN models (AlexNet, GoogleNet, Inception V4, Inception ResNet V2 and ResNeXt-50) using weights (This is a random value of initial weights for our pre-trained CNN architectures). The aforementioned CNN architecture takes advantage of hyper-parameters for DTL, as shown in Table 2.

### 4.3. Results and Analysis

The proposed methodology in this work was evaluated using the performance metrics (see Section 3.6), which were calculated during experiments. This methodology also explores the fine-tuning of DTL, which includes the extraction of the features from pretrained CNN networks. The experimental study was conducted using our custom dataset which is completely based on 4 publicly available datasets as described in Section 1.4. The complete experimental process was based on five pre-trained CNN networks, i.e., AlexNet, GoogleNet, Inception V4, Inception ResNet V2 and ResNeXt-50.

The process starts by evaluating the accuracy for a multi-class dataset. Next, k-fold is utilized to evaluate the average classification accuracy. The value of the average accuracy is calculated using the values of individual accuracy. The results illustrate the exploration of the DTL method using feature extraction on pre-trained CNN networks. The results are listed in Table 3 using our custom dataset (The performance of the experiments was obtained using finetuned and pre-trained architectures for all k-folds). It is noteworthy that the CNN classifies the images and reports a confusion matrix for each severity level of disease.

Table 3 for different k-folds indicates the accuracy value for the employed pre-trained models. The most accurate ResNeXt-50 model is 97.53% for fold-5 (fold-1 and fold-2 also reach the same accuracy). It is clear that the accuracy of the model increases when we increase the value of the fold. Our unique data package achieves the highest precision of 95.98% for fold-5 and the lowest precision of 84.01% for fold-1 for AlexNet models. The same precision is achieved by fold-3, fold-4 and fold-5, for Alexnet and GoogleNet. As regards the maximum individual accuracy of the pretrained models, we have AlexNet: 87.84%, GoogleNet: 91.16%, V4: 90.31%, ResNet V2: 91.61% and ResNeXt-50: 97.53%. For all AlexNet and GoogleNet models pretrained we observe that V4, ResNet V2 and ResNeXt-50 achieve comparable accuracy after fold-3; GoogleNet after fold-1, changing after fold-5; ResNet V4 after fold-1, changing after fold-5; ResNet V2 for fold-2 and fold-3; and ResNeXt-50 for fold-1, fold-2 and fold-5.

### 4.4. Comparison and Discussion

The comparison of our evaluation with the recent publications is presented in Table 4. We found two recent publications and analyzed our implementation results. The authors in [15] evaluated Alexnet and ResNet architectures using a small dataset. This collection includes 4476 pictures from three clinical departments of the Sichuan Provincial People’s Hospital. The authors in [11] implemented Inception V4 and ResNeXt-50 architectures with a dataset that is moderate in size. They utilized a pre-processing pipeline that converts a set of fundus images into a uniform format. They used a modified version of the Inception-V3 network. Subsequently, the performance is compared with several mainstream CNN models. The authors in [11] classified the dataset into five different classes with AlexNet and ResNet. The utilized dataset of 35,126 images exercised three types of ensembles.

In our work, we implemented several mainstream CNN architectures and evaluated five different parameters with DTL using our custom data. The authors in [24] exploited a modified version of Lenet-5 architectures that is quite old. However, in our study, we exploited recent CNNs. Regarding evaluation scores, the proposed methodology efficiently classifies images in different classes as DR, normal, mild, moderate, severe and high-risk. From our analysis, we conclude that the fine-tuning of a pre-trained CNN architecture with DTL could be employed as one of the efficient techniques in the medical field for the classification of DR images.

It is noteworthy that high-risk DR patients lie in the proliferate category. They require an immediate cure and diagnosis. In our diagnosis method, we exploit DR images of patients that reflect the posterior pole. Using a high resolution ultimately elevates the size of the dataset, which may increase the execution time for the classification. However, the use of low-resolution DR images could affect classification due to the lack of clear media. In this research work, we used high-resolution datasets from different sources as mentioned in Section 1.4. To handle this issue, we executed pre-processing of data where the DR images were resized and augmentation was performed for the picture enhancement. Last but not least, there is still a possibility of an error during classification that could be reduced, but the accuracy of the dataset reflects the correctness of the diseases. From the comparison of the state-of-the-art, we conclude that our datasets achieve better accuracy and this was the main goal of this research work.

## 5. Conclusions and Future Work

In this article we have provided a deep-transfer-learning technique, based on convolutional neural networks, for the categorization of diabetic retinopathy patients. In order to investigate the proposed deep-transfer-learning technique, five pre-trained convolutional neural network models were employed. It was observed that the fine tweaking of pre-trained models may be used effectively on a multi-class dataset. As a result, the diagnosis efficiency for diabetic retinopathy patients has been improved. Across all the five employed models, the ResNeXt-50 architecture achieved a maximum accuracy of 97.53 % for our dataset. Our high-accuracy findings can assist doctors and researchers in making clinical judgments. Our work includes a few limitations that can be addressed in future research. A more in-depth examination is needed, which requires more patient data. Future study should also focus on differentiating the accuracy of individuals with normal symptoms from those with non-proliferate symptoms. The non-proliferate symptoms may not be properly visible on retina images, or may not be visualized at all. Another probable direction is to apply the proposed method to larger datasets. It may address other medical problems such as cancer and tumors, as well being applicable in other computer vision industries such as energy, agriculture and transportation.

## Figures and Tables

**Table 1:** Different stages of diabetic retinopathy with the passage of time [9].

Stage	Normal	Non-Proliferate			Proliferate
Years	0	3–5	5–10	10–15	>15
Type of DR	N/A	Mild	Moderate	Severe	High-risk
Condition of retina	Healthy	A few tiny bulges in the blood vessels	Little lumps in the veins with noticeable spots of blood spillage that stores the cholesterol.	Larger areas of blood leakage. Beading in veins that is unpredictable. The formation of new blood vessels at the optic circle. Vein occlusion.	High bleeding and the formation of new blood vessels elsewhere in the retina. Complete blindness.

**Table 2:** The setting of parameters for CNN architectures.

Parameters	AlexNet	GoogleNet	Inception V4	Inception ResNet V2	ResNeXt-50
Optimizer	ADAM	ADAM	ADAM	ADAM	ADAM
Base learning rate	1e^-5^	1e^-5^	1e^-5^	1e^-5^	1e^-5^
Learning decay rate	0.1	0.1	0.1	0.1	0.1
Momentum $\beta_{1}$	0.9	0.9	0.9	0.9	0.9
RMSprop $\beta_{2}$	0.999	0.999	0.999	0.999	0.999
Dropout rate	0.5	0.5	0.5	0.5	0.5
# of epochs	30	30	30	30	30
Train batch size	32	32	32	32	32
Test batch size	8	8	8	8	8
Total number of parameters	60 M	4 M	43 M	56 M	27.56 M

**Table 3:** Results and performance obtained using pre-trained CNN architectures.

Classifier	Folds	TP	TN	FP	FN	Accuracy (%)	Specificity (%)	Precision (%)	Recall (%)	Fscore (%)
**AlexNet**	F1	37	210	35	12	84.01	85.71	51.38	75.51	61.15
	F2	38	210	37	12	83.50	85.02	50.66	76.0	60.80
	F3	38	214	27	8	87.80	88.79	58.46	82.60	68.46
	F4	37	216	27	8	87.84	88.88	57.81	82.22	67.89
	F5	37	216	27	8	87.84	88.88	57.81	82.22	67.89
**GoogleNet**	F1	38	219	22	7	89.86	90.87	63.33	84.44	72.38
	F2	40	222	19	7	90.97	92.11	67.79	85.10	75.47
	F3	38	221	18	8	90.87	92.46	67.85	82.61	74.51
	F4	37	220	18	8	90.81	92.43	67.27	82.22	74.00
	F5	38	220	18	7	91.16	92.43	67.85	84.44	75.24
**Inception V4**	F1	39	224	21	7	90.37	91.42	65.00	84.78	73.58
	F2	39	224	17	8	91.32	92.94	69.64	82.97	75.72
	F3	39	225	16	8	91.66	93.36	70.90	82.97	76.47
	F4	39	226	18	8	91.06	92.62	68.42	82.98	75.00
	F5	39	222	20	8	90.31	91.73	66.10	82.98	73.58
**Inception ResNet V2**	F1	40	220	18	6	91.55	92.44	68.96	86.96	76.92
	F2	40	221	14	6	92.88	94.04	74.07	86.96	80.00
	F3	40	227	14	7	92.71	94.19	74.07	85.11	79.21
	F4	41	226	13	5	93.68	94.56	75.92	89.13	82.00
	F5	39	223	18	6	91.61	92.53	68.42	86.67	76.47
**ResNeXt-50**	F1	41	233	8	5	95.47	96.68	83.67	89.13	86.31
	F2	41	234	7	5	95.82	97.09	85.41	89.13	87.23
	F3	42	234	6	4	96.50	97.50	87.50	91.30	89.36
	F4	42	236	5	3	97.20	97.92	89.36	93.33	91.30
	F5	41	236	5	2	97.53	97.92	89.13	95.35	92.13

**Table 4:** Comparison with state-of-the-art classifiers.

Classifiers	Alexnet			Inception V4			ResNet/ResNeXt-50		
	Acc (%)	Pre (%)	Rec (%)	Acc (%)	Pre (%)	Rec (%)	Acc (%)	Pre (%)	Rec (%)
Our Work	87.84	57.81	82.22	90.31	66.10	82.98	97.53	89.13	95.35
S. Kumar et al. [11]	60.10	–	–	–	–	–	55.70	–	–
Z. Gao et al. [15]	–	–	–	88.72	95.77	9484	87.61	95.76	95.52

**Acc:** Accuracy, **Pre:** Precision, **Rec:** Recall.

## Data Availability

Not applicable.
